# Temporal Progression of Entry Factors into the Vicious Circle of Dry Eye in Untreated Sufferers

**DOI:** 10.3390/life14070806

**Published:** 2024-06-26

**Authors:** Jacobo Garcia-Queiruga, Hugo Pena-Verdeal, Belen Sabucedo-Villamarin, Carlos Garcia-Resua, Maria J. Giraldez, Eva Yebra-Pimentel

**Affiliations:** 1 GI-2092 Optometry, Departamento de Física Aplicada, Facultad de Óptica y Optometría, Universidade de Santiago de Compostela, Campus Vida s/n, 15701 Santiago de Compostela, Spain; belen.sabucedo@rai.usc.es (B.S.-V.); carlos.garcia.resua@usc.es (C.G.-R.);; 2 AC-24 Optometry, Instituto de Investigación Sanitaria de Santiago de Compostela (IDIS), Travesía da Choupana, 15701 Santiago de Compostela, Spain

**Keywords:** dry eye disease, long-term follow-up, tear meniscus height, lipid layer pattern, bulbar hyperemia

## Abstract

Background: Dry eye disease (DED) is characterized by the loss of ocular surface homeostasis with specific signs and symptoms. Studying the progression of a multifactorial disease is exceedingly challenging for researchers because several factors can influence it. The present study aims to study changes in tear meniscus height (TMH), lipid layer pattern (LLP), and bulbar hyperemia over time in untreated DED participants. Methods: This retrospective longitudinal study included 73 participants (146 eyes) diagnosed with DED since at least 2013. Participants underwent new examinations between 2021 and 2023, grouped by 8-, 6-, or 4-year follow-up periods. TMH, LLP, and bulbar hyperemia were assessed in both examinations. No participant received pharmacological treatment for DED. Results: Differences in TMH, bulbar hyperemia, and LLP between sessions were obtained in the 8-year group (*p* ≤ 0.027). Differences in bulbar hyperemia and LLP between sessions were obtained in the 6-year group (*p* ≤ 0.022). The only differences in LLP between sessions were obtained in the 4-year group (*p* < 0.005). Conclusion: Changes in TMH were obtained after periods of eight years from the first eye examination. Also, changes in bulbar hyperemia were obtained at periods of 8 and 6 years; however, changes in LLP could be found from 4-year follow-ups.

## 1. Introduction

Dry eye disease (DED) is a multifactorial and chronic condition, characterized by the loss of ocular surface homeostasis, that shows specific signs and symptoms [1]. Due to actual lifestyle habits, there has been an increase in the prevalence range of DED, varying from 8.7% to 42.0% based on symptoms and signs and from 5.0% to 50.0% when studies include symptomatic participants regardless of DED signs [2,3]. Lifestyle has an important implication for the prognosis and progression of the disease, which could be influenced by many triggers related to physical factors (obesity, eye rubbing, pregnancy, etc.), mental health problems (depression, anxiety, sleep disorders, etc.), and social factors (smoking, cannabis, recreational drugs, etc.) [4]. DED could result from just one factor or the combination of various factors, which are also related to physical triggers such as contact lens wear or the use of video display terminals for prolonged periods of time, such as during work and/or leisure time, among others [5,6,7,8]. Overall, patients should undergo specific DED assessments to understand the actual state of the disease by completing specific questionnaires to evaluate the presence of symptoms and performing specific ocular tests validated for the diagnosis of DED, such as tear film break-up time, ocular surface staining (corneal, bulbar, or palpebral conjunctiva), or osmolarity [9].

To date, the natural course of the disease is still unknown, but several hypotheses have been proposed due to the knowledge of the vicious circle of the disease [10,11]. The main hypothesis is that a patient may dive into this vicious circle due to desiccating stress on the ocular surface originating from hyperosmolarity [10]. Once this occurs, a series of compensatory events may attempt to regulate the hyperosmolarity by increasing tear production or blinking frequency. A patient could have more than one entry point into the vicious circle of the disease due to the different etiologies of DED [11,12]. The most prevalent type of DED is its evaporative form, known as Evaporative Dry Eye (EDE), which is characterized by entering the vicious circle due to alteration in the lipid layer of the tear film [1]. However, when tear production is reduced, the type is known as Aqueous Deficient Dry Eye (ADDE). This form is often linked to Sjögren’s syndrome, but it can also occur independently [1,13]. In both cases, the treatment options focus on reducing ocular surface desiccation and friction to restore ocular homeostasis, with the instillation of artificial tear drops being the main treatment option [12,14].

Understanding the evolution or changes over time of a multifactorial disease is a big challenge for researchers around the world, as the condition is influenced by ethnicity, environmental factors, and other variables [2,3,4,7,8]. To date, numerous short-term studies were conducted in which patients underwent treatment across sessions with short temporal spacing [15,16]. While these studies provide valuable insights into immediate treatment responses and short-term disease dynamics, there are very few previous analyses in the literature that exclusively focus on patients who do not adhere to the recommended treatments and are followed over long periods. This gap in the literature is significant because non-adherence to treatment is common in clinical practice, and understanding the long-term progression of diseases in these patients can lead to improved management strategies and patient education. Indeed, the study of changes in ocular parameters may be undertaken by scheduling patients at different time periods; however, the challenge lies in getting them to return over extended periods [17]. Furthermore, it is necessary to have a strict and clinically and scientifically validated classification system to prevent the emergence of biases during these longitudinal evaluations that may disrupt the actual evolution and change that may be occurring. The Tear Film and Ocular Surface Society (TFOS) in the Dry Eye Workshop II (DEWS II) recommended some specific tests for properly classifying patients into the ADDE or EDE groups, such as measurement of tear meniscus height (TMH) or lipid layer patterns (LLP), respectively [9,18]. Furthermore, the measurement of additional parameters such as conjunctival or bulbar hyperemia could improve the estimation of the inflammatory status associated with DED severity [19]. This present study aims to study changes in TMH, LLP, and bulbar hyperemia over different time periods in untreated DED participants.

## 2. Materials and Methods

### 2.1. Study Design

The present manuscript reflects a retrospective longitudinal study as each included participant received an initial diagnosis of DED due to signs and symptoms compatible with the disease since at least 2013. During the period from 2021 to 2023, a new ocular examination was performed to assess how ocular parameters change over time. This fact segregated the samples into 3 groups since the first examination: 8-year follow-up, 6-year follow-up, and 4-year follow-up. In both examinations, TMH, LLP, and bulbar hyperemia were evaluated, besides the standard procedures for diagnosing DED, including ocular symptomatology, osmolarity, tear film break-up time, and corneal staining [9]. In all tests, the data were pseudonymized with alphanumeric masking to prevent identification during analysis by a second observer.

### 2.2. Sample

All participants were diagnosed with DED by their medical doctor from the Medical Service of the institution, who referred them to the Optometry Clinic for an ocular examination. To be included in the study, no participant could have undergone any kind of pharmacological treatment for DED; they were only instructed to follow some visual indications to improve their symptomatology (e.g., 20-20-20 rule). The 20-20-20 rule is a popular and effective recommendation for the users of video display terminals who exhibit symptoms of DED [20]. This guideline advises that after every 20 min of continuous screen use, individuals should look away for at least 20 s, focusing on something at least 20 feet away (6 m). A diagnosis of DED was confirmed in all the participants at the Optometry Clinic, following the TFOS DEWS II diagnostic guidelines [9,18]. To be diagnosed with DED, participants must present symptoms of DED and at least one ocular sign of homeostasis alteration. The presence of DED symptoms was determined by administering the Ocular Surface Disease Index (OSDI) questionnaire, with a score of ≥12 indicating the presence of DED [21,22]. The presence of ocular homeostasis alterations was determined by showing corneal staining ≥ 1, tear film break-up time < 10 s, or osmolarity values > 308 mOsm/L [9,18]. Also, participants were excluded if they had a history of ocular surgery, active blepharitis, glaucoma, ocular allergy, or systemic disorders that could affect the ocular surface directly or due to their pharmacological treatment, or if they were pregnant or breastfeeding [23].

The sample size was calculated using the PS Power and Sample Size Calculations 3.1.2 software (Copyright© by William D. Dupont and Walton D. Plummer). The TFOS DEWS II Diagnostic Methodology report was used as a reference; therefore, the Standard Deviations (SDs) of TMH values were assumed to be 0.05 mm [9,24]. To achieve a statistical power of 80% (Type II error associated) at a significance level of α = 0.05 (Type I error associated) and a confidence level of 95% in detecting a clinical difference between participants without pathology and those with pathology, a minimum of 13 subjects was determined per group. These values are in concordance with the recommendations of the TFOS DEWS II Diagnostic Methodology [9].

### 2.3. Procedures

All the procedures were performed using the same instruments and protocol in both examinations, regardless of the follow-up group to which the participants were assigned. In all sessions, tests were conducted in the same order, from the least to the most invasive, in both participants’ eyes [9]. The protocol was carried out under controlled environmental conditions of light, temperature (20–23 °C), and humidity (50–60%). Every participant signed a written informed consent to be included in the study. The present study adhered to the tenets of the Declaration of Helsinki and was approved by the Bioethics Committee of the institution (USC-40/2020). A second blind observer randomly analyzed all measurements for each procedure.

#### 2.3.1. Tear Meniscus Height

TMH was measured using the illumination of the Tearscope interferometer (Keeler, Windsor, UK) attached to a Topcon SL-D4 (Topcon Corporation, Tokyo, Japan) slit lamp. The Tearscope is a clinical tool for assessing lipid layer thickness using a cylindrical cool white fluorescent light source, which produces unique observations specific to this illumination [25]. All measurements were videorecorded using the Topcon DC-4 (Topcon Corporation, Tokyo, Japan) attached to the slit lamp, a video camera that allows clinicians to capture videos and images of the ocular surface. Once the Tearscope was properly positioned in front of the objective of the slit lamp, the participant was instructed to look to the center of the device’s illumination. Throughout the recording, illumination was provided by the Tearscope with the slit lamp turned off; the brightest setting of the two illumination options provided by the device was always employed to ensure optimal limit definition and visualization. A video of the lower meniscus was captured from both eyes of each participant involved in the study. From the recorded videos, a frame where the entire meniscus was in focus was obtained for TMH calculation. To avoid interblink variations, images were consistently extracted 2–3 s after blinking, when the meniscus was stable, under minimal changes, and fully expanded. Central TMH, defined as the distance between the end of the darker edge of the lower eyelid and the upper limit of the tear meniscus, was assessed using the open-source software ImageJ software v1.54f (National Institutes of Health, Bethesda, MD, USA), as previous reports have described [26].

#### 2.3.2. Lipid Layer Pattern

The Tearscope was previously mounted onto the SL-D4 slit lamp and was used to illuminate the ocular surface, generating an interference pattern of the lipid layer on the muco-aqueous layer of the tear film, known as LLP [27,28]. Similar to the previous protocol, the Tearscope was fixed to the slit lamp, maintaining a constant distance between the chinrest and the device to ensure a consistent imaging area. Illumination was provided by the Tearscope, and all videos met minimum quality standards, being free of blurring, well centered, and with the lipid layer well spread after a complete blink. A video of LLP was captured from both eyes of each participant involved in the study. The LLP videos were then analyzed and classified according to Guillon’s scheme. Guillon’s scheme classifies LLP videos into the following categories [28]: Grade 1—Open Meshwork, Grade 2—Close Meshwork, Grade 3—Fluid, Grade 4—Amorphous, and Grade 5—Colored. The categories correspond to the estimation of the thickness of the lipid layer from the lowest to the highest thickness.

#### 2.3.3. Bulbar Hyperemia

Images of the bulbar conjunctiva for measuring ocular hyperemia were captured using the DC-4 video camera attached to the SL-D4 slit lamp. Participants were instructed to look to the right and left to capture images of the nasal and temporal bulbar conjunctiva. A video was captured from both eyes of each participant involved in this study. Based on those videos, two images were extracted: one from the nasal bulbar conjunctiva and one from the temporal bulbar conjunctiva. All images were graded following the Brien Holden Vision Institute (BHVI) scheme [29]. The BHVI grading scale categorizes bulbar hyperemia into 4 severity grades: Grade 1—Very Slight, Grade 2—Slight, Grade 3—Moderate, and Grade 4—Severe. The overall bulbar hyperemia of each eye was obtained by averaging the nasal and temporal bulbar hyperemia.

### 2.4. Statistical Analysis

IBM SPSS Statistics for Windows, Version 25.0 (IBM Corp., Armonk, NY, USA) was used for the data analysis, and significance was set at a *p* ≤ 0.05 for all analyses. The normal distribution of the data was tested for each procedure in each group [30]. Depending on the sample size, either the Kolmogorov–Smirnov or Shapiro–Wilk tests were used. Descriptive statistics, including the mean, standard deviation, median, and interquartile range, were calculated to summarize the data for each ocular parameter across the different follow-up periods. Furthermore, the Wilcoxon signed-rank test for repeated samples was performed because all the procedures of each group followed a non-parametric distribution (Kolmogorov–Smirnov or Shapiro–Wilk test, all *p* ≤ 0.05) [30].

## 3. Results

A total of 146 eyes were included in the present study, distributed as follows: 61 eyes in the 8-year follow-up group (78.8% woman, 50.1 ± 8.8 years old), 30 in the 6-year follow-up group (61.1% woman, 47.4 ± 11.3 years old), and 55 in the 4-year follow-up group (72.7% woman, 50.6 ± 10.28 years old). The participants of each group attended the second session after 2919.2 ± 21.0 days, 2206.1 ± 39.5 days, and 1471.6 ± 23.6 days, respectively. Comparisons were made where data permitted, and so the sample analyzed in each procedure was represented in the table for each group.

### 3.1. Analysis of Differences between Sessions in the 8-Year Follow-Up Group

**Figure 1:**
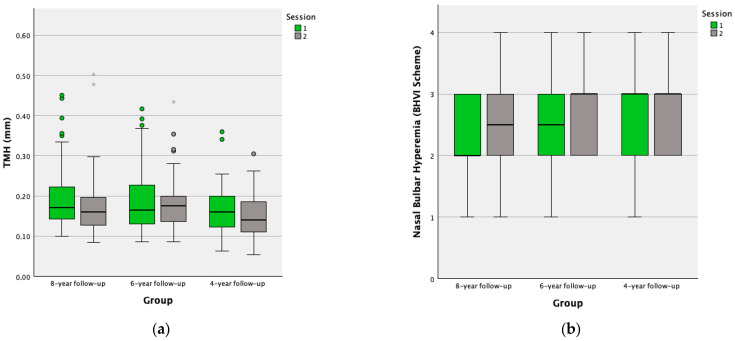

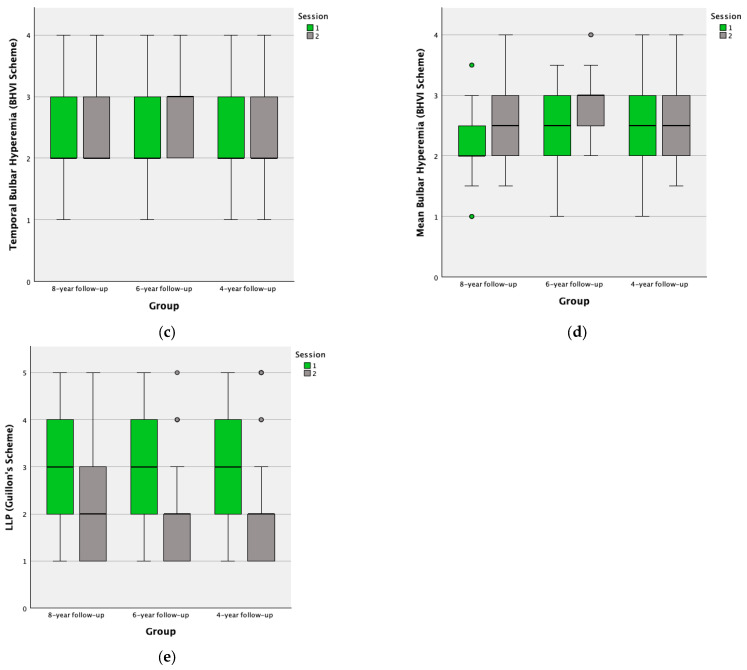
Boxplots for the different ocular parameters studied on each follow-up group and each session: (**a**) TMH Boxplot; (**b**) Nasal Bulbar Hyperemia Boxplot; (**c**) Temporal Bulbar Hyperemia Boxplot; (**d**) Mean Bulbar Hyperemia Boxplot; (**e**) LLP Boxplot. The box represents the sample between the interquartile range (25th–75th percentile) and the black line represents the median value. The dots represent outliers (values more than 1.5 box lengths away from the 25th or 75th percentile) and the asterisk represents extreme outliers (values more than 3 box lengths away from the 25th or 75th percentile). TMH = tear meniscus height; LLP = lipid layer pattern; BHVI = Brien Holden Vision Institute.

Table 1 shows the descriptive statistics and differences between session 1 and session 2 in the 8-year follow-up group. All the studied parameters showed differences between sessions (Wilcoxon test, all *p* ≤ 0.027): TMH decreased, nasal and temporal bulbar hyperemia increased, and LLP decreased (Figure 1).

### 3.2. Analysis of Differences between Sessions in the 6-Year Follow-Up Group

Table 2 shows the descriptive statistics and differences between session 1 and session 2 in the 6-year follow-up group. Bulbar hyperemia (nasal, temporal, and mean of both) and LLP showed differences between session 1 and session 2 (Wilcoxon test, all *p* ≤ 0.022): all hyperemia results increased while LLP decreased (Figure 1). On the contrary, no statistical difference between sessions was observed for TMH (Wilcoxon test, *p* = 0.165) (Figure 1).

### 3.3. Analysis of Differences between Sessions in the 4-Year Follow-Up Group

Table 3 shows the descriptive statistics and differences between session 1 and session 2 in the 4-year follow-up group. No statistical differences between sessions were observed for most parameters (Wilcoxon test, all *p* ≥ 0.189) (Figure 1). However, a statistical difference was obtained between session 1 and session 2 for LLP (Wilcoxon test, *p* < 0.001), showing a decrease in the value between sessions (Figure 1).

## 4. Discussion

Multifactorial diseases have different pathogenic factors that influence the origin or establishment of the disease. In the case of DED, various factors may always be present, like the alteration of tear film stability, epithelial damage of the ocular surface, and clinical inflammation [12]. These factors could also serve as entry points to the pathophysiological vicious circle of the disease, but they could originate from different events. To date, there has been a scarce number of studies that describe the natural course of the untreated DED [17,31]. Previous reports have proposed a theoretical model that outlines three stages of DED progression (initiation, reflex compensation, and the loss of compensatory response); this model suggests that the disease may deteriorate without intervention and over time, with a possible plateau at certain stages [32]. Despite the theoretical models, there has been a notable lack of clinical studies that specifically focus on the long-term progression of DED in untreated patients [17,33,34]. Most existing research has not set untreated patient follow-up as a primary objective, leaving a gap in the understanding of how the disease evolves without intervention. It is necessary to study these processes in parts, analyzing how each parameter or tear film characteristic could be affected by the vicious circle. This gap highlights the need for comprehensive longitudinal studies to better understand DED’s natural history and develop more effective management strategies.

Tear film stability is an ocular parameter influenced by a wide range of alterations, such as changes in the lipid layer of the tear film or a decrease in the total tear volume [35]. In the present study, the 8-year and 6-year follow-up groups showed that, after those periods, the values in TMH and LLP were reduced. Nevertheless, in the 4-year follow-up group, LLP was reduced too, but TMH did not change. Several reports have studied the reproducibility of the TMH or LLP, but no one has evaluated these parameters in long-term studies like the present manuscript. Measuring the TMH is a useful procedure to quantify or estimate the total tear volume in the ocular surface [36]. Bilkhu et al. [37] studied the evaporative pathophysiology of DED by exposing the participants to a situation that stresses the ocular surface, playing a high-concentration game on a tablet computer for 30 min. After that task, TMH values were statistically and significantly decreased, and dry eye symptomatology was enhanced, showing that certain activities could lead to joining the vicious circle of DED. Continuous exposure to high-concentration activities commonly present in a day-to-day task is responsible for originating the disease. Nevertheless, Li et al. [38] measured the tear meniscus volume by OCT over eight hours and observed no change in either the DED sample or the normal sample. Considering previous studies and the results of the present report, it can be hypothesized that changes in TMH values over time are not detectable in periods shorter than 8 years. This is evidenced by the lack of significant differences observed in participants examined at the 4-year and 6-year intervals.

Meibomian glands are intrinsically related to the LLP because these structures are responsible for the production of the meibum, which is the main component of the lipid layer. Pult obtained a relationship between age and meibomian gland loss, where elderly subjects showed higher values of loss [39]. Once the DED is established, and after periods of 8-year, 6-year, and 4-year, there can be an important loss of meibomian glands in the sample managed by the present study, reflected in the values obtained in LLP. Lower values of LLP indicate a thinner lipid layer that could originate from the malfunction of the meibomian glands, related to their destruction or loss. Subjectively grading the LLP following the Guillon scheme may not differentiate small changes in lipid layer thickness; however, the measurement of LLP following this technique was able to differentiate changes in all follow-up groups studied in the present report [40]. Future studies could use other instruments to measure the thickness of the lipid layer in microns, such as LipiView II [41], in order to assess the state of the meibomian glands and determine whether there is a relationship between them after prolonged periods of disease.

The main sign indicating inflammation of the ocular surface is ocular hyperemia. Ocular hyperemia can occur acutely when blood flow increases near the corneo-scleral limbus, indicating the presence of a pathogen in the corneal tissue [42]. However, when hyperemia occurs throughout the bulbar conjunctiva, it is a chronic inflammatory process that affects the entire ocular surface. Previous reports have found higher values of conjunctival hyperemia in EDE subjects with severe dry eye symptoms than in those with less symptomatology [43]. In the present study, a statistically significant increase in bulbar hyperemia values was observed from the first to the second session in the 8-year and 6-year follow-up groups. Long-term follow-up seems to be the key to understanding the period after which the disease worsens since the 4-year follow-up group showed no differences between sessions.

This study has several notable strengths that provide strong scientific results on the natural course of the disease. First, it follows the standardized diagnostic criteria of the TFOS DEWS II guidelines [18], ensuring consistency and reliability in identifying and classifying the disease and avoiding bias in the inclusion of preclinical or predisposed DED participants [7]. Moreover, the longitudinal design employed allows for the observation of changes in ocular parameters in an unbiased recruited sample over extended periods, aiding in the understanding of the natural progression of untreated DED. In addition, the present study also includes a blinded analysis of the data, reducing bias and increasing the objectivity of the results.

Nevertheless, despite the valuable insights provided by the study, there were certain limitations. First, the follow-up involves three unrelated groups observed over different periods, offering useful information. However, future research could enhance our understanding by tracking the same group across various time points. Secondly, the variation in sample sizes among the studied groups was due to lost measurements during the eye examinations, which resulted from instrument malfunctions or other uncontrollable factors. Future research should control for every factor that could affect the sample size of any group. Third, this study exclusively explores the natural progression of untreated dry eye disease, potentially limiting the generalizability of the findings to this specific population.

## 5. Conclusions

In conclusion, in the present study, it was observed that changes in TMH could be detected after periods of 8 years from the first eye examination. On the other hand, changes in bulbar hyperemia were obtained at periods of 8 and 6 years; however, changes in LLP values could be found from a 4-year follow-up. The observed differences among follow-up groups underscore the heterogeneity of DED, emphasizing the necessity for customized approaches to patient management. Future research should delve into the mechanisms driving these temporal variations and assess targeted interventions to delay disease progression, ultimately enhancing therapeutic strategies for improved patient outcomes.

## Figures and Tables

**Table 1:** Descriptive statistics and differences between sessions in the 8-year follow-up group.

Ocular Parameter		N	Session	Mean ± SD	Median (IQR)	*p*-Value
TMH (mm)		61	1	0.20 ± 0.79	0.18 (0.15–0.23)	0.006 *
			2	0.18 ± 0.79	0.17 (0.13–0.20)	
Bulbar hyperemia (BHVI Scheme)	Nasal	34	1	2.18 ± 0.67	2 (2–3)	0.010 *
			2	2.59 ± 0.60	3 (2–3)	
	Temporal	34	1	2.21 ± 0.81	2 (2–3)	0.027 *
			2	2.53 ± 0.71	2 (2–3)	
	Mean	34	1	2.19 ± 0.65	2 (1.88–2.50)	0.003 *
			2	2.56 ± 0.56	2.50 (2–3)	
LLP (Guillon’s Scheme)		38	1	3.13 ± 1.32	3 (2–4)	0.016 *
			2	2.50 ± 1.20	2 (2–3)	

* Statistically significant (*p* < 0.05). TMH = tear meniscus height; LLP = lipid layer pattern; SD = standard deviation; IQR = interquartile range; BHVI = Brien Holden Vision Institute.

**Table 2:** Descriptive statistics and differences between sessions in the 6-year follow-up group.

Ocular Parameter		N	Session	Mean ± SD	Median (IQR)	*p*-Value
TMH (mm)		30	1	0.20 ± 0.09	0.18 (0.13–0.26)	0.165
			2	0.19 ± 0.08	0.18 (0.14–0.20)	
Bulbar hyperemia (BHVI Scheme)	Nasal	30	1	2.47 ± 0.82	3 (2–3)	0.022 *
			2	2.83 ± 0.65	3 (2–3)	
	Temporal	30	1	2.37 ± 0.67	2 (2–3)	0.007 *
			2	2.80 ± 0.71	3 (2–3)	
	Mean	30	1	2.42 ± 0.66	2.50 (2–3)	0.002 *
			2	2.82 ± 0.61	3 (2.38–3.13)	
LLP (Guillon’s Scheme)		30	1	2.93 ± 1.34	3 (2–4)	0.009 *
			2	2.17 ± 1.09	2 (1–2.25)	

* Statistically significant (*p* < 0.05). TMH = tear meniscus height; LLP = lipid layer pattern; SD = standard deviation; IQR = interquartile range. BHVI = Brien Holden Vision Institute.

**Table 3:** Descriptive statistics and differences between sessions in the 4-year follow-up group.

Ocular Parameter		N	Session	Mean ± SD	Median (IQR)	*p*-Value
TMH (mm)		51	1	0.16 ± 0.61	0.16 (0.11–0.20)	0.189
			2	0.15 ± 0.05	0.14 (0.11–0.18)	
Bulbar Hyperemia (BHVI Scheme)	Nasal	55	1	2.56 ± 0.66	3 (2–3)	0.382
			2	2.66 ± 0.67	3 (2–3)	
	Temporal	55	1	2.35 ± 0.70	2 (2–3)	0.714
			2	2.38 ± 0.71	2 (2–3)	
	Mean	55	1	2.45 ± 0.59	2.50 (2–3)	0.601
			2	2.52 ± 0.62	2.50 (2–3)	
LLP (Guillon’s Scheme)		54	1	3.04 ± 1.30	2 (2–4)	<0.001 *
			2	2.26 ± 1.17	2 (1.75–3)	

* Statistically significant (*p* < 0.05). TMH = tear meniscus height; LLP = lipid layer pattern; SD = standard deviation; IQR = interquartile range. BHVI = Brien Holden Vision Institute.

## Data Availability

Data is unavailable due to privacy restrictions.
